# Evaluation of Polycyclic Aromatic Hydrocarbons Content and Risk Assessment of Tea Products in South Korea

**DOI:** 10.3390/foods14091530

**Published:** 2025-04-27

**Authors:** Kyung-Jik Lim, Yoon-Hee Lee, Han-Seung Shin

**Affiliations:** Department of Food Science and Biotechnology, Dongguk University-Seoul, 32, Dongguk-ro, Ilsandong-gu, Goyang-si 10326, Gyeonggi-do, Republic of Korea; kyung9209@naver.com (K.-J.L.); dldbsgml491@naver.com (Y.-H.L.)

**Keywords:** polycyclic aromatic hydrocarbon (PAH), tea, tea infusions, toxic equivalency (TEQ), margin of exposure (MOE)

## Abstract

This study investigated the levels of four polycyclic aromatic hydrocarbons (BaA, CHR, BbF, and BaP) in 11 types of 100 commercially available tea products using gas chromatography–mass spectrometry (GC-MS), and also evaluated potential dietary risks, toxic equivalency (TEQ), and margin of exposure (MOE). Method validation demonstrated strong linearity of the calibration curves for all four PAHs (*R*^2^ > 0.99) over a concentration range of 1–20 μg/kg. The LOD for the four PAHs ranged from 0.0610 to 0.1534 μg/kg in the solid matrix and from 0.0035 to 0.0064 μg/kg in the liquid matrix, with corresponding LOQ ranging from 0.1849 to 0.4648 μg/kg in the solid matrix and from 0.0107 to 0.0194 μg/kg in the liquid matrix. All recovery rates were within the acceptable range, demonstrating satisfactory performance, and both intraday and interday accuracy and precision were within acceptable limits, meeting international validation criteria. Among the samples, yerba mate tea (33.58 μg/kg), herbal tea (24.05 μg/kg), and oolong tea (23.21 μg/kg) showed the highest Σ4PAH concentrations. Based on these results, TEQ_BaP_ and MOE values were calculated for the positive samples. All three teas with detectable PAHs exhibited MOE values above 10,000, indicating a low level of potential carcinogenic risk. However, the presence of PAHs in certain tea types highlights the importance of ongoing monitoring, regulatory oversight, and risk communication to ensure consumer safety.

## 1. Introduction

Tea, the second most consumed beverage globally after water, is enjoyed by nearly three billion people daily. However, tea remains susceptible to polycyclic aromatic hydrocarbon (PAH) contamination through several pathways, including environmental pollution and their migration from packaging materials. Furthermore, PAHs can be generated during cooking, and their levels in the cooked food are influenced by factors such as the food type, the cooking method, and the fuel used [1].

PAHs are a group of organic compounds widely present in the environment, and they are characterized by molecular structures consisting of two or more fused benzene rings [2]. These compounds form during incomplete combustion or decomposition of organic materials at high temperatures, commonly in biomass burning or fossil fuel use [3,4]. Benz[a]anthracene (BaA), chrysene (CHR), benzo[b]fluoranthene (BbF), and benzo[a]pyrene (BaP) are recognized as carcinogenic and genotoxic PAHs and collectively known as the 4PAH group [1]. These compounds are identified by both the European Food Safety Authority (EFSA) and the International Agency for Research on Cancer (IARC) as primary markers of PAH contamination in food. The IARC classified BaP as a Group 1 carcinogen, signifying an unequivocal cancer risk to humans. BaA, CHR, and BbF were categorized as Group 2A or 2B carcinogens [5]. Due to their carcinogenic and genotoxic properties, PAHs are closely regulated in food products, particularly in items like tea, grilled meats, oils, and smoked products, where environmental exposure and processing can elevate PAH levels, aiming to reduce health risks from dietary exposure [6].

According to Roudbari et al. [7], the minimum mean concentration of PAHs and the mean concentration of BaP in tea samples were 4.77 ± 1.01 µg/kg and ranged from 0.64 to 2.07 µg/kg, respectively. Due to the large surface area of tea leaves, PAHs can accumulate, particularly from the air. They can also be introduced during production, as some tea leaves are dried using combustion gases from burning wood, oil, or coal. These gases always contain PAHs, which can be absorbed upon contact [8].

Given the potential health risks, it is crucial to implement effective food processing techniques and safety management practices to minimize PAH contamination in tea. This study focuses on evaluating PAH contamination in 100 commercially available tea products in South Korea, including both tea leaves and brewed infusions. The toxic equivalency (TEQ) and margin of exposure (MOE) values were calculated to assess potential health risks. By quantifying PAHs and conducting dietary risk assessments, this study provides practical data for improving PAH monitoring and regulatory practices in the tea industry.

## 2. Materials and Methods

### 2.1. Chemicals and Materials

The 4PAH standards, namely BaA (CAS No. 56-55-3), CHR (CAS No. 218-01-9), BbF (CAS No. 205-99-2), and BaP (CAS No. 50-32-8) were acquired from Sigma-Aldrich (St Louis, MO, USA). Internal standards (ISs), such as CHR-d12 (CAS No. 1719-03-5) and BaP-d12 (CAS No. 63466-71-7), were obtained from Sigma-Aldrich. All chemicals used in the study were of analytical grade. Water (CAS No. 7732-18-5), dichloromethane (DCM; CAS No. 75-09-2), and n-hexane (CAS No. 110-54-3) were procured from Honeywell International Inc. (Charlotte, NC, USA); ethyl alcohol (CAS No. 64-17-5) came from Samchun Pure Chemical Co., Ltd. (Pyeongtaek, Republic of Korea); and N,N-dimethylformamide from Daejung Chemicals & Metals Co., Ltd. (Si-heung, Republic of Korea). Potassium hydroxide (KOH, CAS No. 1310-58-3) was employed for the saponification process. Sodium sulfate (Na2SO4, CAS No. 7757-82-6) from Daejung Chemicals & Metals Co., Ltd., was used as a drying agent. For solid-phase extraction (SPE) and sample purification, Sep-Pak Florisil cartridges from Waters Corp. (Milford, MA, USA) were used. PTFE membrane filters (0.45 μm) were obtained from Agilent Technologies, Inc. (Santa Clara, CA, USA). A total of 100 tea products were collected from the local market in Gyeonggi-do, Republic of Korea, representing 11 different types of tea: Cassia tora tea (*n* = 9), green tea (*n* = 9), buckwheat tea (*n* = 8), barley tea (*n* = 10), black tea (*n* = 9), Solomon’s seal tea (*n* = 8), yerba mate tea (*n* = 10), corn tea (*n* = 10), brown rice tea (*n* = 9), herbal tea (*n* = 9), and oolong tea (*n* = 9). The teas were tested in their finished-product form, either as teabags or solid tea leaves, depending on the product. Among these, green tea, black tea, and oolong tea are made from the leaves of the tea plant (*Camellia sinensis*), while the others are made from different plants or herbs, such as *Cassia tora, Polygonatum odoratum,* and *Ilex paraguariensis.* The herbal tea samples were primarily composed of chamomile (*Matricaria chamomilla*), a commonly consumed herbal infusion.

### 2.2. Sample Preparation

For the analysis of solid tea products, tea leaves were ground using a grinder equipped with stainless-steel blades (Model SMX-P400DKP, Manufacturer Sin-Il, Seoul, Republic of Korea), ensuring uniform particle size suitable for analytical procedures. The grinding process using the mixer produced a fine powder with an estimated particle size of approximately 180 μm. A total of 10 g of the ground tea product was used for subsequent analysis.

To ensure consistency in the water quality used across all samples, 2 g of each tea product was infused in 400 mL of boiling distilled water for 5 min. Brewing was conducted in heat-resistant glass beakers, and the resulting infusions were filtered using a 0.45 μm membrane filter to remove particulates. From the filtered tea infusion, 10 g of the solution was used for further analysis after cooling to room temperature.

### 2.3. Extraction and Clean-Up

The analytical procedure for solid tea leaf samples was adapted from the general test method for “non-fatty solid matrix” established by the Ministry of Food and Drug Safety (MFDS) of Korea [9]. For solid tea leaf samples, 10 g of the homogenized material was accurately weighed into a 300 mL round-bottom flask and spiked with 1 mL of an internal standard solution containing 100 μg/kg of chrysene-d_12_ (CHR-d_12_) and benzo[a]pyrene-d_12_ (BaP-d_12_). The internal standard concentration of 100 μg/kg was chosen to provide measurable signals within the instrument’s linear range while effectively compensating for matrix effects during the analysis. To facilitate alkaline hydrolysis and promote the release of PAHs from the matrix, 100 mL of 1 M potassium hydroxide (KOH) in ethanol was added, and the mixture was refluxed at 80 °C for 3 h. After cooling to room temperature, 50 mL of n-hexane and an additional 50 mL of a 1:1 (*v*/*v*) mixture of ethanol and n-hexane were added. The mixture was filtered and transferred to a separatory funnel, followed by two sequential extractions with 50 mL of n-hexane. The combined organic layers were washed three times with 50 mL of distilled water to remove polar impurities.

The upper n-hexane layer was collected, dried over anhydrous sodium sulfate (Na_2_SO_4_), and transferred to a 250 mL round-bottom flask. The extract was concentrated at 37 °C using a rotary evaporator. For further purification, solid-phase extraction (SPE) was conducted using Sep-Pak Florisil cartridges (6 cc Vac Cartridge, 50–200 µm). The cartridges were sequentially eluted with 5 mL of n-hexane, followed by 15 mL of a 3:1 (*v*/*v*) mixture of n-hexane and dichloromethane (DCM). The eluate was evaporated to dryness under a gentle stream of nitrogen at 37 °C and reconstituted in 1 mL of DCM. Prior to analysis, the solution was filtered through a 0.45 μm PTFE membrane filter and transferred to amber vials (2 mL). A 1 μL aliquot was injected into a gas chromatography–mass spectrometry (GC–MS) system for quantification.

For liquid samples, approximately 10 g of the homogenized sample was accurately weighed and spiked with 1 mL of an internal standard solution containing 100 μg/kg of CHR-d_12_ and BaP-d_12_. Then, 100 mL of n-hexane was added, and the mixture was vigorously shaken and subjected to ultrasonic extraction for 30 min. The extract was transferred to a separatory funnel, and after phase separation, the aqueous layer was extracted twice with 50 mL of n-hexane. The combined organic layers were washed twice with 50 mL of distilled water. If emulsions formed, 5–10 mL of methanol was added to resolve them, and the aqueous phase was discarded. The hexane layer was dried over anhydrous sodium sulfate, filtered, and concentrated to approximately 2 mL under reduced pressure at temperatures below 40 °C. The purification procedure was identical to that used for the solid samples.

### 2.4. Determination of PAHs by GC-MS

The extracted samples were analyzed using an Agilent Technologies 7820A/5975 MSD GC-MS system (Santa Clara, CA, USA). Separation was achieved using a Zebron ZB-PAH-SeleCT column (30 m × 0.25 mm, 0.20 µm particle size), specifically designed for selective and efficient separation of PAHs. Sample volumes of 1.0 μL were injected in the splitless mode at an injection temperature of 320 °C, which ensured optimal volatilization and transfer of PAH analytes onto the column without thermal degradation. The column temperature was initially held at 80 °C for 1 min and then gradually raised to 245 °C, at a rate of 6 °C/min, to achieve effective resolution of early-eluting compounds. Subsequently, the temperature was further increased to 270 °C, at a rate of 30 °C/min, and maintained for 13 min to allow complete elution of relatively higher-molecular-weight PAHs. A post-run temperature of 310 °C was maintained for 10 min to eliminate residual contaminants and prepare the column for subsequent analyses. Ultra-high-purity helium (99.99%) was employed as the carrier gas, maintaining a stable flow rate of 1.2 mL/min, thus providing consistent chromatographic performance and reproducible retention times. The mass spectrometer operated in electron ionization (EI) mode with selected ion monitoring (SIM), optimizing detection sensitivity and selectivity for targeted PAH compounds. The ion source and quadrupole temperatures were set at 250 and 150 °C, respectively, to enhance analyte ion stability and analytical precision.

### 2.5. Validation of the Analytical Method for PAHs

The method was validated according to Codex Alimentarius guidelines (CAC/GL-71) for key parameters, including accuracy, precision, linearity, limit of detection (LOD), and limit of quantification (LOQ). Validation was performed using brown rice tea, and calibration curves were established at five concentrations: 1, 2, 5, 10, and 20 μg/kg. The validation process involved testing two different matrices: non-fatty solid and non-fatty liquid. Calibration curves were established by injecting 1.0 μL of each standard mixture in triplicate. LOD is the lowest concentration of a substance that can be detected but not quantified. It was calculated based on a signal-to-noise ratio (S/N) of 3.3. LOQ is the lowest concentration at which a substance can be quantitatively measured with acceptable precision and accuracy. It was calculated based on a S/N of 10. Accuracy and precision were evaluated through repeated analysis of spiked samples, with intraday analysis performed three times on the same day and interday analysis conducted on three separate days.

### 2.6. Risk Assessment and Toxic Equivalency Evaluation

A risk assessment was performed while following the guidelines set by the U.S. Environmental Protection Agency (EPA), using the toxic equivalency factors (TEFs) outlined in the EPA’s provisional guidance for evaluating polycyclic aromatic hydrocarbons (PAHs) [10]. TEF is a coefficient used to assess the relative toxicity of specific compounds within groups of chemically similar substances, such as PAHs, dioxins (PCDD/Fs), and polychlorinated biphenyls (PCBs).

In this study, the toxic equivalency of PAH mixtures was evaluated using the benzo[a]pyrene toxic equivalency quotient (TEQ_BaP_). The TEQ_BaP_ serves as an indicator of the total carcinogenic potential of PAH mixtures by comparing their cumulative toxicity to that of benzo[a]pyrene (BaP), a well-established reference compound with a TEF value of 1.

The TEQ_BaP_ for each tea infusion sample was calculated using the following equation:

TEQ_BaP_ = ∑[C_i_] × TEF_i_

where C_i_ is the concentration of PAH congener i in the tea sample (µg/kg), and TEF_i_ is the toxic equivalency factor of congener i, as defined by the EPA. Each PAH concentration was multiplied by its corresponding TEF, and the results were summed up to obtain a unified toxicity estimate for each sample.

The EDI was calculated to evaluate daily exposure to toxic PAHs through tea consumption, using TEQ_BaP_ values obtained for each sample. EDI represents the amount of toxic PAHs ingested per unit of body weight per day and was calculated using the following formula:

EDI (ng/kg bw/day) = C × IR/BW

where C is the TEQ_BaP_ concentration in tea infusion (ng/mL), IR is the average daily intake of tea (300 mL/day), and BW is the average adult body weight (60 kg). This standardized approach allows for the quantification of internal exposure under typical consumption conditions.

MOE was calculated to assess the potential carcinogenic risk associated with PAH intake, based on the estimated EDI values. MOE was determined using the following equation:

MOE = BMDL_10_/EDI

where BMDL_10_ is the benchmark-dose lower confidence limit for a 10% increase in tumor incidence, set at 70,000 ng/kg bw/day according to EFSA guidelines [6].

### 2.7. Statistical Analysis

All experiments were performed in triplicate, and the results are presented as the mean ± standard deviation (SD). To determine significant differences (*p* < 0.05), one-way ANOVA and Duncan’s multiple range test were applied. All data were statistically analyzed using IBM SPSS Statistics, version 27 (Armonk, NY, USA).

## 3. Results and Discussion

### 3.1. Validation and Analytical Quality Assurance for Four PAHs’ Analysis

**Figure 1:**
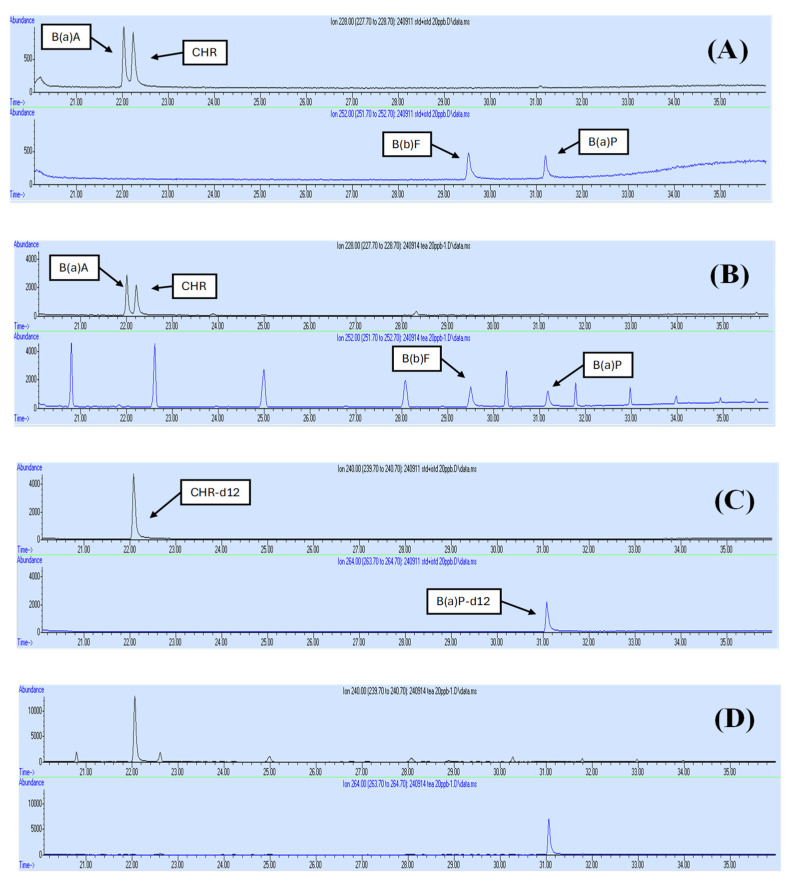
GC-MS chromatograms of B(a)A, CHR, B(b)F, and BaP standards: (**A**) blank sample with 4PAH standards, (**B**) spiked sample containing 4PAH standards, (**C**) internal standards in a blank sample, and (**D**) spiked sample containing internal standards.

**Figure 2:**
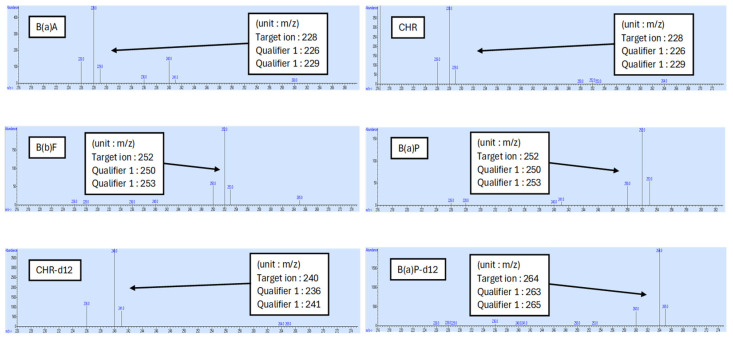
Mass spectra of 4PAH standards and two internal standards.

The GC-MS chromatograms of the standards of BaA, CHR, BbF, and BaP in a blank sample and a spiked sample containing the 4PAHs are shown in Figure 1. The mass spectra of the individual standards of each of the 4PAHs and two ISs are depicted in Figure 2.

Calibration curves were created by analyzing the response factors of the PAHs relative to the IS at five different concentrations (1, 2, 5, 10, and 20 μg/kg). Each standard solution contained 100 µg/kg of an IS and was spiked into the sample. Recovery rates, linearity, LOD, and LOQ were subsequently evaluated, with results presented in Table 1. The calibration curves, which correlate the chromatographic peak areas with the concentrations, showed strong linearity across all concentrations, with R^2^ values exceeding 0.99. The LODs for the 4PAHs in the solid matrix ranged from 0.0610 to 0.1534 μg/kg, with corresponding LOQs ranging from 0.1849 to 0.4648 μg/kg. In the liquid matrix, the LODs ranged from 0.0035 to 0.0064 μg/kg, and the LOQs ranged from 0.0107 to 0.0194 μg/kg. The recovery of the 4PAHs from the tea matrix showed satisfactory performance, falling within an acceptable range, as detailed in Table 2.

Interday accuracy and precision were evaluated by carrying out three replicate analyses on the same day, maintaining consistent experimental conditions across all trials to assess the consistency and reproducibility of the results within a single day. By conducting both intraday and interday evaluations, the overall robustness and reliability of the analytical technique could be established, ensuring that the method could produce reliable results across both short and extended periods. For the solid matrix, intraday and interday accuracy ranged from 80.84% to 107.92% and from 80.46% to 107.26%, respectively, while precision ranged from 0.06% to 1.03% for intraday and from 0.74% to 12.62% for interday. For the liquid matrix, intraday and interday accuracy ranged from 99.99% to 100.02% and from 98.86% to 101.48%, respectively, with precision ranging from 0.00% to 0.37% for intraday and from 0.06% to 1.32% for interday. The detailed accuracy and precision values for the 4PAHs are shown in Table 3. The analytical methods demonstrate satisfactory linearity, detection and quantification limits, recovery, and precision, fulfilling the validation criteria outlined in the Codex Alimentarius guidelines (CAC/GL-71).

### 3.2. Comparison of PAHs Concentrations Between Tea Leaves and Brewed Tea

The concentrations of each of the 4PAHs, along with their total concentration (Σ4PAHs), were measured in 100 tea samples, and the results are summarized in Table 4. All results are expressed as the mean and SD based on three replicate analyses. In the tea samples, the mean concentrations of BaA, CHR, BbF, and BaP were 2.53, 4.86, 1.18, and 0.66 μg/kg, respectively.

PAH concentrations were consistently higher in solid tea leaves than in their brewed infusions. This discrepancy can be attributed to the physicochemical properties of PAHs, particularly their low aqueous solubility and hydrophobicity. High-molecular-weight PAHs, such as benzo[a]pyrene (BaP) and benzo[b]fluoranthene (BbF), possess four or more fused aromatic rings, which significantly reduce their solubility in water and limit their transfer from solid matrices into aqueous solutions during typical brewing conditions (hot water at~90–100 °C) [6,11]. Numerous studies have demonstrated that the solubility of PAHs in water is inversely correlated with their molecular weight and number of aromatic rings [12]. For example, BaP has an aqueous solubility of approximately 3.8 μg/L at 25 °C, while lighter PAHs, such as naphthalene, are much more soluble (~30,000 μg/L) [13]. As a result, even when PAHs are present in tea leaves at quantifiable levels, their migration into brewed infusions remains limited. Furthermore, strong interactions between PAHs and plant matrix components, such as lipids, proteins, or cell wall structures, may further hinder their extraction into water-based solvents [14]. These findings emphasize the necessity of analyzing both the solid form and aqueous infusions of tea to comprehensively assess human exposure to PAHs via tea consumption. They also highlight the importance of considering solubility and matrix binding effects when interpreting the health risks associated with thermally generated food contaminants.

The overall mean concentration of the Σ4PAHs in the tea samples was 9.23 μg/kg. Yerba mate tea exhibited the highest average concentration of BaP, at 2.49 μg/kg, with the Σ4PAH concentration reaching 33.58 μg/kg, also the highest across all analyzed samples. This may be attributed to its traditional drying process, which is commonly reported to involve burning wood, oil, or coal—known sources of PAHs that can be absorbed by tea leaves. Yerba mate leaves also have a large surface area, promoting the adsorption of particle-bound PAHs from the air [15]. According to previous studies [16,17], yerba mate is processed through two distinct thermal treatments that are believed to contribute to PAH formation: the sapeco phase, in which freshly harvested leaves are briefly exposed to high temperatures to inactivate enzymes and prevent oxidation, and a subsequent drying phase involving prolonged heat exposure to reduce moisture content. These descriptions are literature-based and were not empirically verified in this study. According to a previous study, yerba mate leaves contained an average Σ4PAH concentration of 872.6 μg/kg and had the highest BaP levels among leaf matrices [18]. Ilex paraguariensis, commonly known as yerba mate, has been reported in previous studies to contain total concentrations of 21 PAHs ranging from 536 to 2906 ng per gram of dried leaves. The concentration of benzo[a]pyrene ranged from 8.03 to 53.3 ng per gram of dried leaves [15]. Additionally, mate leaves are often subjected to smoking during drying, which can lead to high levels of PAHs in commercial products [19].

Herbal tea was found to have the second-highest concentration of Σ4PAHs, measuring 24.05 μg/kg. The BaP level in herbal tea was 2.29 μg/kg. It is important to note that herbal teas encompass a broad category of beverages derived from various plant species. Therefore, when analyzing PAH content, this diversity should be taken into account. According to other studies, chrysene (CHR) was identified as the most abundant compound among the four key PAHs (BaP, BaA, CHR, BbFA) in all tested dried tea samples, which is consistent with our finding that CHR was the highest in herbal tea [20]. According to a previous study, Moringa oleifera infusion is a popular beverage among herbal tea consumers, and the concentration of ∑10PAHs in the dried leaves of 10 brands of M. oleifera ranged from 1.06 to 5.51 µg/kg [21]. In a study of various herbal teas sold in Romania, based on the limits set by Commission Regulation (EU) 2023/915 for BaP and Σ4PAHs [22], one sample exceeded the regulatory threshold for Σ4PAHs, and five samples exceeded the allowable limit for BaP [23]. Another study reported that the highest concentration of Σ16PAHs in green, herbal, and black tea products sold in Nigeria, 79.61 μg/kg, was found in Sahul Slim Herbal Tea, a herbal tea produced in India, and the lowest concentration was observed in Kidney Flush Tea, at 4.71 μg/kg. Results showed that the comparatively higher PAH levels in Sahul Slim Herbal Tea may be linked to the presence of numerous additives in the product, each of which contributes to the overall PAH content [24].

Oolong tea had a similar total 4PAH concentration of 23.21 μg/kg to that of herbal tea, with BaP detected at 1.19 μg/kg. Furthermore, CHR levels in oolong tea were recorded at 13.31 μg/kg, representing the second-highest CHR concentration among all tea samples. The Camellia sinensis leaves used in oolong tea have a very large surface area, making them more susceptible to contamination by PAHs and other harmful compounds [25]. A previous study showed that the Σ13PAHs concentration was higher in oolong tea (110 ng/g) compared to green tea (83.3 ng/g) [26]. This difference is believed to stem from the distinct processing techniques used because, in contrast to green tea, which is either steamed or pan-fried to prevent oxidation [27], oolong tea undergoes sun wilting, bruising, and partial fermentation of fresh leaves.

Black tea also showed a notable Σ4PAH content of 14.24 μg/kg, with CHR reaching 6.99 μg/kg, a higher value compared to other tea samples. This result indicates a relatively high PAH concentration in black tea. Although the specific processing method for each sample could not be confirmed—since all samples were purchased from the domestic Korean market—the elevated levels suggest the possibility of PAH introduction during certain stages of production, such as drying or through indirect exposure to combustion products. Especially, black tea is fully fermented before undergoing processes like smoking, during which it may absorb PAHs from the smoke generated by burning wood [28]. Previous studies also reported that black tea contained the highest BaP and Σ4PAH levels among tested teas, with BaP ranging from 4.1 to 32 μg/kg and Σ4PAHs ranging from 25 to 115 μg/kg [29]. These findings support the need for further investigations into the processing conditions of commercial black teas and their potential impact on PAH levels.

It is important to note that the descriptions of tea processing methods presented in this section, including those for yerba mate, herbal, oolong, and black teas, were inferred from the prior literature and not empirically verified in this study. Therefore, any association between processing techniques and PAH levels should be interpreted with caution.

Green tea showed a Σ4PAH content of 2.04 ± 0.39 μg/kg, with BaA and CHR reaching 0.68 ± 0.10 μg/kg and 1.36 ± 0.30 μg/kg, respectively. According to previous studies, during the “fixing” step of green tea production, the tea leaves are roasted on a metal plate at 180–220 °C, resulting in significant water loss and the potential formation of PAHs due to the thermal degradation of organic matter [30]. Green tea is produced by first withering the leaves, followed by steaming or pan-firing to inactivate enzymes, then drying, grading, and packaging without undergoing fermentation [28]. Although fermentation is omitted, this thermal processing provides conditions conducive to PAH formation. Additionally, tea leaves, with their extensive surface area, are particularly prone to accumulating PAHs, especially from airborne sources [8]. Therefore, the presence of PAHs in green tea can be attributed to a combination of both environmental exposure and thermal processing.

The concentrations of BaP in P. odoratum tea, green tea, corn tea, C. tora tea, buckwheat tea, barley tea, and brown rice tea were below the LOQ. These teas, all classified as non-fermented, tend to exhibit lower levels of detectable PAH compounds compared to their fermented counterparts. Previous studies suggest that non-fermented teas, such as green tea, generally contain lower PAH concentrations, which may be attributed to the absence of high-temperature processing or fermentation steps that could facilitate PAH formation, in contrast to fully fermented teas like black tea, which undergo processes that may enhance PAH formation during production [31,32].

### 3.3. Risk Assessment

The TEF values are provided in Table 5. After applying these values to all tea products with BaP as the reference, the concentrations of BaP ranged from 0.00 to 2.49 μg TEQ_BaP_/kg, and the 4PAHs varied from 0.00 to 3.82 μg TEQ_BaP_/kg. The TEF calculation enabled a standardized evaluation of each PAH’s contribution to the overall toxicity, revealing significant variations in contamination levels among the different types of tea. Yerba mate tea exhibited TEQ_BaP_ values of BaP and 4PAHs of 2.49 and 3.82 μg/kg, respectively. These values were the highest observed across all tea samples. Herbal tea was the second highest, with a BaP toxic equivalent of 2.29 μg TEQ_BaP_/kg and a 4PAH toxic equivalent of 3.63 μg TEQ_BaP_/kg. Oolong tea samples showed a BaP toxic equivalent of 1.19 μg TEQ_BaP_/kg, and the 4PAH toxic equivalent reached 2.19 μg TEQ_BaP_/kg, indicating relatively high levels among the samples analyzed. Black tea had a BaP toxic equivalent of 1.12 μg TEQ_BaP_/kg, with a 4PAH toxic equivalent of 1.80 μg TEQ_BaP_/kg.

The PAH analysis data for tea infusions are summarized in Table 6. While PAHs were detected in certain tea extracts, the PAH levels in green tea, barley tea, C. tora tea, buckwheat tea, P. odoratum tea, corn tea, black tea, and brown rice tea were all below the quantification limit. The levels of PAHs detected in infusions were relatively low for yerba mate tea, herbal tea, and oolong tea. Previous research indicates that a decrease in the tea-to-water ratio (TWR) is associated with a reduction in PAH concentration in tea infusions. This is likely due to the greater dispersion of tea compounds in water at lower TWR values, resulting in a dilution of PAH concentrations [33].

To assess the potential health risks from PAH exposure through tea consumption, the present study calculated the MOE by analyzing PAH concentrations in brewed tea infusions. Using EFSA (2008) guidelines [6] and considering a per capita tea consumption of 0.9 kg and an average body weight (bw) of 51.09 kg, the MOE was calculated based on the benchmark-dose lower confidence limits (BMDL_10_) for BaP (0.07 mg/kg bw/day) and the 4PAHs (0.34 mg/kg bw/day). Table 7 presents the margin of exposure (MOE) values for BaP and four polycyclic aromatic hydrocarbons (PAHs) in various tea samples, providing an overview of the PAH exposure levels across different tea types and assessing the safety of typical consumption amounts. The PAH concentrations in the infusions were used for the calculation in four different tea types, offering an overview of PAH exposure margins in various tea varieties and highlighting the safety of typical consumption levels.

The EFSA guidelines state that MOE values approaching or below 10,000 may suggest potential health concerns [6]. However, in the present study, the MOE values ranged from 329,000 to 535,000 for BaP and from 227,000 to 484,000 for the 4PAHs, suggesting that the health risks associated with tea consumption are minimal. According to a previous study, based on the average dietary exposure for adult consumers in China, the chronic-exposure MOE values for BaP, Σ2PAHs, Σ4PAHs, and Σ8PAHs in all analyzed tea samples were 144,970.4, 55,674.7, 43,492.9, and 54,775.1, respectively. Additionally, the chronic- and acute-exposure MOE values for all six types of tea consumed by adults in the Beijing–Tianjin–Hebei region of China were found to be within the safe range (MOE ≥ 10,000), leading to the conclusion that PAH intake from tea does not pose a serious health risk to humans [34]. A study evaluating the transfer rate of PAHs from leaves to infusions found that the calculated MOE values for 2PAHs and 4PAHs exceeded 10,000 in all cases, indicating a low level of concern for human health, and this served as a benchmark for assessing the potential impact on human health [35].

This study has several limitations that warrant consideration when interpreting the results. First, although the analytical method was validated, potential matrix effects resulting from the complex chemical composition of tea samples may have affected the accuracy and precision of PAH quantification. Second, the relatively small number of samples per tea type (approximately 8–10) may limit the statistical power and generalizability of the findings to the broader population of commercially available tea products. Third, due to the commercial origin of the samples, detailed information regarding their manufacturing processes—such as specific drying conditions or the type of fuel used—was not available. As a result, the associations between processing techniques and PAH levels discussed in this study are based solely on the existing literature and were not directly verified through empirical observation. Therefore, further studies employing controlled processing conditions and larger, more representative sample sets are necessary to validate and expand upon these findings.

### 3.4. PAH Source Attribution by Diagnostic Ratios

The BaA/(BaA + CHR) ratio, a commonly used diagnostic tool, was calculated to evaluate the potential sources of PAH contamination in solid tea samples. This ratio is widely employed to differentiate between petrogenic sources (typically < 0.2), biomass combustion (0.2–0.35), and strong pyrogenic sources associated with high-temperature thermal processes (>0.35) [36,37,38,39,40]. As presented in Table 8, the diagnostic ratios varied considerably depending on the type of tea, providing insight into the likely origin of PAHs in each case.

As shown in Table 8, the BaA/(BaA + CHR) ratios varied significantly across different tea types. Yerba mate tea exhibited a ratio of 0.28, suggesting partial contribution from combustion-related sources, which may be associated with traditional drying methods involving the burning of wood or coal. PAHs can be absorbed into tea leaves during traditional drying processes involving the combustion of wood, oil, or coal, and yerba mate leaves are known to be particularly susceptible to adsorbing airborne particulate PAHs due to their large surface area [15]. Herbal tea showed a higher ratio of 0.47, reflecting a pronounced influence from combustion sources, possibly due to the use of various plant-based ingredients and mixed processing conditions that may introduce combustion byproducts. A previous study reported BaA/(BaA + CHR) ratios of 0.40, 0.33, 0.32, and 0.17 in several herbal teas, indicating that vehicular emissions, biomass combustion, and petrogenic sources may contribute to PAH accumulation in some herbal teas [24]. Oolong tea and black tea showed BaA/(BaA + CHR) ratios of 0.34 and 0.32, respectively. Both types of tea undergo fermentation and drying processes during production, during which traditional fuels such as wood, oil, or coal may be used. If so, the tea leaves can be directly exposed to combustion byproducts, increasing the likelihood of PAHs being absorbed [15]. Barley tea showed an extreme value of 1.00, which resulted from the detection of BaA only without CHR. Such a value should be interpreted cautiously, as it may reflect either selective PAH presence or a result of analytical detection limits.

In contrast, Cassia tora tea, buckwheat tea, Polygonatum odoratum tea, corn tea, and brown rice tea showed a ratio of 0.00 or could not be calculated, indicating that neither BaA nor CHR was detected. These results suggest that PAH contamination in these samples is minimal or absent. The use of the BaA/(BaA + CHR) ratio thus provides a quantitative approach to understanding the potential origin of PAHs in various tea samples and can aid in evaluating the influence of raw materials, drying conditions, and fuel types used in processing.

Future studies should extend this approach by applying multiple diagnostic ratios across a broader range of PAHs and by incorporating detailed processing information or controlled model experiments. Such efforts would contribute to a more comprehensive understanding of PAH contamination sources and support the development of effective mitigation strategies in tea manufacturing.

## 4. Conclusions

This study examined the health risks posed by PAHs in tea infusions, focusing on their concentrations and calculating the MOEs for BaP and 4PAHs using the BMDL_10_ values. Eleven tea types were analyzed, including yerba mate, herbal, and oolong teas. While higher PAH levels were detected in some raw tea materials, the PAH levels in the brewed tea infusions, which represent the form in which tea is commonly consumed, were found to be low.

For estimating daily PAH intake, the MOE values were determined. The results showed that the MOE for BaP ranged from 329,000 to 535,000, and the MOE for 4PAHs ranged from 227,000 to 484,000, all of which are significantly above the EFSA safety threshold of 10,000. These high MOE values suggest that the health risks from PAH exposure through tea consumption are minimal, reinforcing the safety of tea consumers regarding PAH contaminants. Given the carcinogenic and genotoxic nature of PAHs, these results are crucial for food safety evaluations. By quantifying PAH contamination in beverages like tea, this research enhances the understanding of potential risks and supports efforts to ensure consumer safety. However, since PAHs can also be ingested through other dietary sources, such as grilled meats, oils, and smoked products, cumulative dietary exposure and potential additive or synergistic effects should be considered. Future studies should aim to assess total PAH intake across multiple food categories, incorporating consumption patterns and interactions between food types to provide a more comprehensive evaluation of dietary risk.

Additionally, potential seasonal or geographical variations in PAH contamination should be considered in future studies. Factors such as climate, agricultural practices, and regional differences in environmental pollution could contribute to fluctuations in PAH levels in tea leaves. Understanding these variations could further inform safety assessments and help mitigate risks associated with PAH exposure in different regions or seasons.

Although only limited amounts of PAHs were transferred to tea infusions, thereby minimizing health risks from tea consumption, it is also important to consider ways to reduce PAH levels in the solid tea materials themselves. Managing the tea manufacturing process is critical in minimizing PAH contamination. In particular, the drying process at high temperatures may lead to incomplete combustion, which can generate PAHs. Lowering the drying temperature and carefully controlling fuel usage can help reduce PAH formation. Therefore, technical improvements that prevent incomplete combustion during tea processing are essential. For example, adjusting drying conditions or applying chemical treatments to lower PAH levels could serve as effective strategies to mitigate the risks associated with PAH contamination in tea. Additionally, adopting alternative drying methods, such as using lower-temperature or electric dryers, could further reduce PAH exposure during production and provide a more sustainable solution for the industry.

## Figures and Tables

**Table 1:** Evaluation of limit of detection (LOD), limit of quantification (LOQ), and linearity of PAH analysis in brown rice tea as a representative sample.

Sample Type	PAHs	Equation (y = ax + b)	Linearity (*R*^2^)	LOD (μg/kg) ^(1)^	LOQ (μg/kg) ^(2)^
Tea leaf (solid)	BaA	y = 0.0096x + 0.0023	0.9992	0.0954	0.2891
	CHR	y = 0.0081x + 0.0016	0.9997	0.0610	0.1849
	BbF	y = 0.0112x + 0.0047	0.9996	0.1440	0.4364
	BaP	y = 0.0077x + 0.0040	0.9997	0.1534	0.4648
Infused tea (liquid)	BaA	y = 0.0079x + 0.0014	0.9995	0.0044	0.0132
	CHR	y = 0.0080x + 0.0010	0.9992	0.0064	0.0194
	BbF	y = 0.0081x + 0.0009	0.9994	0.0037	0.0111
	BaP	y = 0.0080x − 0.0001	0.9991	0.0035	0.0107

^(1)^ Based on a signal-to-noise ratio (S/N) = 3.3. ^(2)^ Based on a signal-to-noise ratio (S/N) = 10.

**Table 2:** Evaluation of recovery rates of PAHs in spiked samples.

Sample Type	4PAHs	Recovery (%) ^(1)^				
		1 (μg/kg)	2 (μg/kg)	5 (μg/kg)	10 (μg/kg)	20 (μg/kg)
Tea leaf (solid)	BaA	87.39 ± 0.48	80.84 ± 0.73	106.64 ± 0.83	105.35 ± 0.48	107.92 ± 0.54
	CHR	86.95 ± 0.70	84.35 ± 0.74	91.55 ± 0.69	88.80 ± 0.38	82.99 ± 0.28
	BbF	98.01 ± 0.74	90.83 ± 0.94	98.74 ± 0.21	103.89 ± 0.21	98.16 ± 0.12
	BaP	107.44 ± 0.07	84.21 ± 0.31	94.04 ± 0.26	100.55 ± 0.19	103.48 ± 0.21
Infused tea (liquid)	BaA	100.02 ± 0.37	99.99 ± 0.08	100.00 ± 0.02	99.99 ± 0.09	100.00 ± 0.00
	CHR	100.02 ± 0.14	99.99 ± 0.05	100.00 ± 0.09	99.99 ± 0.03	100.00 ± 0.07
	BbF	100.00 ± 0.28	100.00 ± 0.11	100.00 ± 0.04	100.00 ± 0.04	100.00 ± 0.05
	BaP	100.00 ± 0.28	100.00 ± 0.06	100.00 ± 0.23	100.00 ± 0.07	100.00 ± 0.03

^(1)^ Recovery was assessed using spiked concentrations of 1, 2, 5, 10, and 20 μg/kg, and the data are presented as mean ± relative standard deviation (*n* = 3).

**Table 3:** Evaluation of accuracy and precision in tea samples across intraday and interday experiments.

Sample Type	4PAHs		Interday (*n* = 3)		Intraday (*n* = 3)	
			Accuracy (%) ^(1)^	RSD (%) ^(2)^	Accuracy (%)	RSD (%)
Tea leaf (solid)	BaA	1	85.20	12.62	87.39	0.55
		2	81.90	2.12	80.84	0.90
		5	105.50	4.97	106.64	0.78
		10	105.63	1.84	105.35	0.45
		20	105.57	4.26	107.92	0.50
	CHR	1	86.75	5.98	86.95	0.81
		2	84.50	5.40	84.35	0.88
		5	91.55	5.29	91.55	0.76
		10	93.31	7.36	88.80	0.43
		20	80.56	3.80	82.99	0.34
	BbF	1	94.39	7.13	98.01	0.75
		2	87.18	5.40	90.83	1.03
		5	97.59	2.29	98.74	0.21
		10	105.10	8.35	103.89	0.20
		20	97.61	0.92	98.16	0.12
	BaP	1	107.26	1.53	107.44	0.06
		2	80.46	4.51	84.21	0.37
		5	96.65	3.01	94.04	0.27
		10	98.04	3.09	100.55	0.19
		20	102.67	0.74	103.48	0.20
Infused tea (liquid)	BaA	1	99.99	0.26	100.02	0.37
		2	100.35	0.35	99.99	0.08
		5	99.77	0.22	100.00	0.02
		10	99.94	0.09	99.99	0.09
		20	100.05	0.06	100.00	0.00
	CHR	1	101.48	1.28	100.02	0.14
		2	100.09	0.10	99.99	0.05
		5	100.20	0.27	100.00	0.09
		10	100.25	0.36	99.99	0.03
		20	100.58	0.65	100.00	0.07
	BbF	1	100.54	0.99	100.00	0.28
		2	100.68	0.69	100.00	0.11
		5	99.79	0.69	100.00	0.04
		10	99.74	0.79	100.00	0.04
		20	99.66	0.42	100.00	0.05
	BaP	1	99.55	0.99	100.00	0.28
		2	101.14	1.31	100.00	0.06
		5	100.51	0.33	100.00	0.23
		10	99.50	0.79	100.00	0.07
		20	98.86	1.32	100.00	0.03

^(1)^ Accuracy (%) = [1 − (mean concentration measured−concentration spiked)/concentration spiked] × 100. ^(2)^ RSD (relative standard deviation%) = (standard deviation/mean) × 100.

**Table 4:** Concentration of four polycyclic aromatic hydrocarbons PAHs in tea samples determined by GC-MS.

Sample	N ^(1)^	BaA (μg/kg)	CHR (μg/kg)	BbF (μg/kg)	BaP (μg/kg)	4PAHs (μg/kg)
Cassia tora tea	9	<LOQ	0.33 ± 0.02	<LOQ	<LOQ	0.33 ± 0.02
Green tea	9	0.68 ± 0.10	1.36 ± 0.30	<LOQ	<LOQ	2.04 ± 0.39
Buckwheat tea	8	<LOQ	0.32 ± 0.03	<LOQ	<LOQ	0.32 ± 0.03
Barley tea	10	0.54 ± 0.06	<LOQ	<LOQ	<LOQ	0.54 ± 0.06
Black tea	9	3.30 ± 0.17	6.99 ± 0.29	2.83 ± 0.19	1.12 ± 0.23	14.24 ± 0.87
Polygonatum odoratum tea	8	<LOQ	0.25 ± 0.02	<LOQ	<LOQ	0.25 ± 0.02
Yerba mate tea	10	7.70 ± 0.72	19.70 ± 1.88	3.69 ± 0.84	2.49 ± 0.46	33.58 ± 3.90
Corn tea	10	<LOQ	0.24 ± 0.03	<LOQ	<LOQ	0.24 ± 0.03
Brown rice tea	9	<LOQ	<LOQ	<LOQ	<LOQ	<LOQ
Herbal tea	9	8.13 ± 0.28	9.32 ± 0.22	4.31 ± 0.34	2.29 ± 0.20	24.05 ± 1.04
Oolong tea	9	6.83 ± 0.64	13.31 ± 1.04	1.87 ± 0.31	1.19 ± 0.29	23.21 ± 2.28

^(1)^ *N*: number of tea samples used for experiment.

**Table 5:** Toxic equivalency BaP TEQ_BaP_ values estimated using toxicity equivalency factors TEFs in dried tea (solid tea leaf) samples.

TEQ_BaP_ Value ^(2)^ (μg-TEQ_BaP_/kg)					
TEF ^(1)^	BaA	CHR	BbF	BaP	4PAHs
	0.10	0.01	0.10	1.00	
Yerba mate tea	0.77	0.20	0.37	2.49	3.82
Herbal tea	0.81	0.09	0.43	2.29	3.63
Oolong tea	0.68	0.13	0.19	1.19	2.19
Black tea	0.33	0.07	0.28	1.12	1.80
Green tea	0.07	0.01	0.00	0.00	0.08
Barley tea	0.05	0.00	0.00	0.00	0.05
Cassia tora tea	0.00	0.00	0.00	0.00	0.00
Buckwheat tea	0.00	0.00	0.00	0.00	0.00
Polygonatum odoratum tea	0.00	0.00	0.00	0.00	0.00
Corn tea	0.00	0.00	0.00	0.00	0.00
Brown rice tea	0.00	0.00	0.00	0.00	0.00

^(1)^ Toxic Equivalency Factor. ^(2)^ Toxic equivalents.

**Table 6:** Migration of PAHs into tea infusion from different tea matrices.

	PAHs Concentrations in the Infusions (μg/kg)				
PAHs	BaA	CHR	BbF	BaP	4PAH
Yerba mate tea	0.0324	0.0756	0.0144	0.0162	0.1386
Herbal tea	0.0395	0.0410	0.0163	0.0195	0.1163
Oolong tea	0.0142	0.0267	0.0121	0.0120	0.0650
Black tea	<LOQ	<LOQ	<LOQ	<LOQ	<LOQ
Green tea	<LOQ	<LOQ	<LOQ	<LOQ	<LOQ
Barley tea	<LOQ	<LOQ	<LOQ	<LOQ	<LOQ
Polygonatum odoratum tea	<LOQ	<LOQ	<LOQ	<LOQ	<LOQ
Corn tea	<LOQ	<LOQ	<LOQ	<LOQ	<LOQ
Cassia tora tea	<LOQ	<LOQ	<LOQ	<LOQ	<LOQ
Buckwheat tea	<LOQ	<LOQ	<LOQ	<LOQ	<LOQ
Brown rice tea	<LOQ	<LOQ	<LOQ	<LOQ	<LOQ

**Table 7:** Margin-of-exposure values for BaP and four polycyclic aromatic hydrocarbons (PAHs) in various tea samples.

Tea Type	BaP		4PAHs	
	Conc. in Tea	MOEs	Conc. in Tea	MOEs
Yerba mate tea	0.0162	397,000	0.1386	227,000
Herbal tea	0.0195	329,000	0.1163	270,000
Oolong tea	0.0120	535,000	0.0650	484,000
Black tea	ND	-	ND	-
Green tea	ND	-	ND	-
Barley tea	ND	-	ND	-
Cassia tora tea	ND	-	ND	-
Buckwheat tea	ND	-	ND	-
Polygonatum odoratum tea	ND	-	ND	-
Corn tea	ND	-	ND	-
Brown rice tea	ND	-	ND	-

**Table 8:** Diagnostic ratios in solid tea samples.

Tea (Solid)	BaA/(BaA + CHR)
Yerba mate tea	0.28
Herbal tea	0.47
Oolong tea	0.34
Black tea	0.32
Green tea	0.33
Barley tea	1.00
Cassia tora tea	0.00
Buckwheat tea	0.00
Polygonatum odoratum tea	0.00
Corn tea	0.00
Brown rice tea	-

## Data Availability

The original contributions presented in this study are included in the article. Further inquiries can be directed to the corresponding author.
